# Cadmium‐Doped Zinc Sulfide Shell as a Hole Injection Springboard for Red, Green, and Blue Quantum Dot Light‐Emitting Diodes

**DOI:** 10.1002/advs.202104488

**Published:** 2022-03-03

**Authors:** Bochen Liu, Yue Guo, Qiang Su, Yunfeng Zhan, Zhao Chen, Yang Li, Baogui You, Xiaonan Dong, Shuming Chen, Wai‐Yeung Wong

**Affiliations:** ^1^ School of Applied Physics and Materials Wuyi University Jiangmen 529020 P. R. China; ^2^ Department of Electrical and Electronic Engineering Southern University of Science and Technology Shenzhen 518055 P. R. China; ^3^ Poly Optoelectronics Tech. Ltd Jiangmen 529020 P. R. China; ^4^ Fujian Science & Technology Innovation Laboratory for Optoelectronic Information of China Fuzhou 350108 P. R. China; ^5^ Department of Applied Biology and Chemical Technology, Research Institute for Smart Energy and Guangdong‐Hong Kong‐Macao Joint Laboratory for Photonic‐Thermal‐Electrical Energy Materials and Devices The Hong Kong Polytechnic University (PolyU) Hung Hom Hong Kong P. R. China; ^6^ PolyU Shenzhen Research Institute Shenzhen 518057 P. R. China

**Keywords:** balanced charge carriers, efficient quantum dot light‐emitting diodes, high photoluminescence quantum yields, hole injection springboard, outermost CdZnS shell

## Abstract

A new strategy is developed in which cadmium‐doped zinc sulfide (CdZnS) is used as the outermost shell to synthesize red, green, and blue (RGB) quantum dots (QDs) with the core/shell structures of CdZnSe/ZnSe/ZnS/CdZnS, CdZnSe/ZnSe/ZnSeS/CdZnS, and CdZnSe/ZnSeS/ZnS/CdZnS, respectively. Firstly, the inner ZnS and ZnSe shells confine the excitons inside the cores of QDs and provide a better lattice matching with respect to the outermost shell, which ensures high photoluminescence quantum yields of QDs. Secondly, the CdZnS shell affords its QDs with shallow valence bands (VBs). Therefore, the CdZnS shell could be used as a springboard, which decreases the energy barrier for hole injection from polymers to QDs to be below 1.0 eV. It makes the holes to be easily injected into the QD EMLs and enables a balanced recombination of charge carriers in quantum dot light‐emitting diodes (QLEDs). Thirdly, the RGB QLEDs made by these new QDs exhibit peak external quantum efficiencies (EQEs) of 20.2%, 19.2%, and 8.4%, respectively. In addition, the QLEDs exhibit unexpected luminance values at low applied voltages and therefore high power efficiencies. From these results, it is evident that CdZnS could act as an excellent shell and hole injection springboard to afford high performance QLEDs.

## Graphical abstract

Red, green, and blue quantum dots (QDs) with the cadmium‐doped zinc sulfide (CdZnS) shell are synthesized. The CdZnS shell affords its QDs with high photoluminescence quantum yields and shallow valence bands, which could act as a hole injection springboard and is beneficial to the hole injection in QD based light‐emitting diodes.

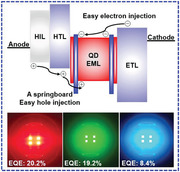

## 1 Introduction

**Figure 1:**
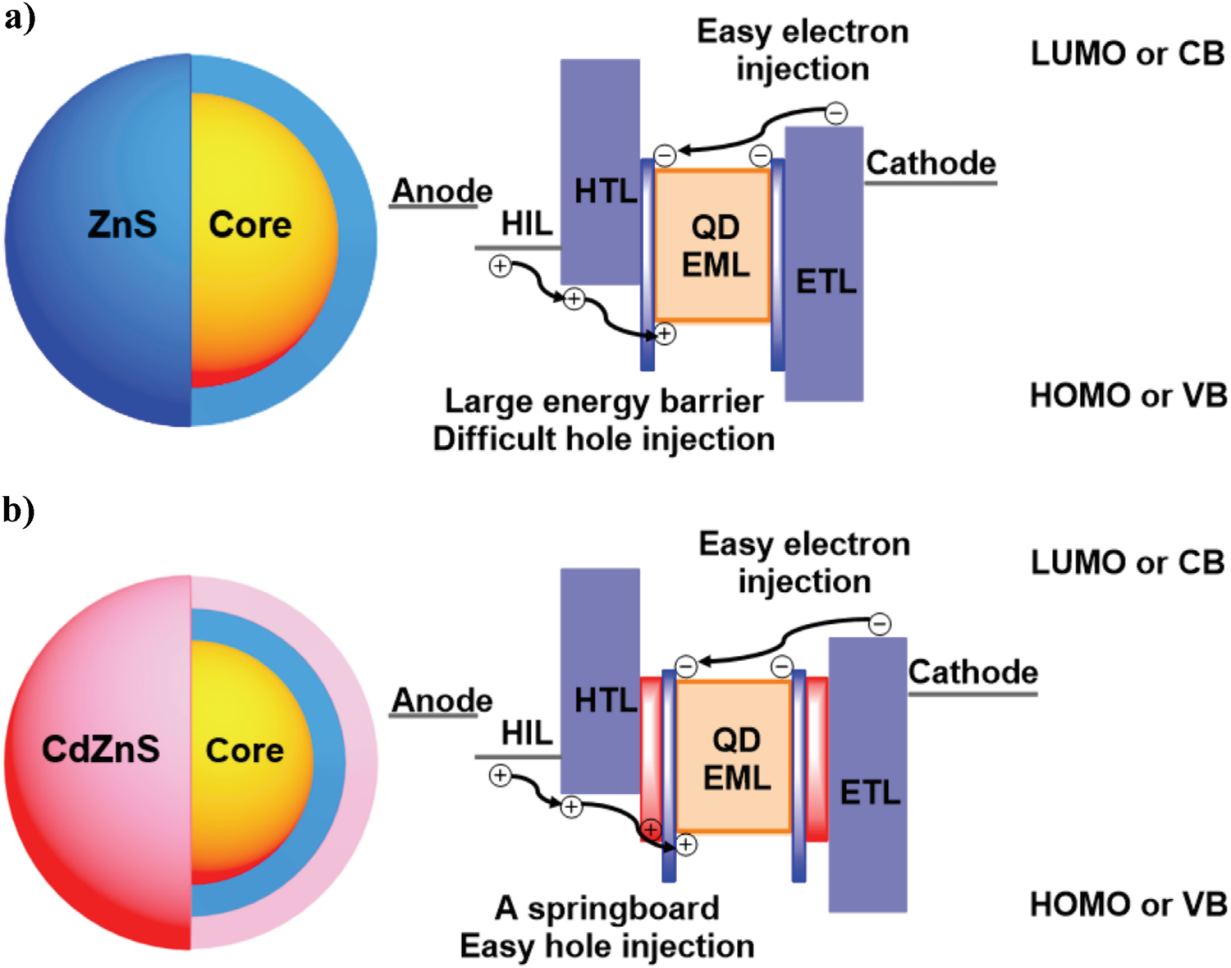
a) A typical type‐II core/shell structure of a QD and an energy diagram in a conventional QLED; b) the design strategy used in this work, in which the outermost cadmium‐doped zinc sulfide (CdZnS) shell is used as a springboard to make hole injection easier (from the core to the outermost shell, the layers with yellow, blue, and red color represent the core, ZnS shell, and outermost CdZnS shell, respectively). LUMO = the lowest unoccupied molecular orbital, CB = conduction band, HIL = hole injection layer, HOMO = the highest occupied molecular orbital.

Due to the high photoluminescence quantum yield (PLQY), tunable emission color that spans the whole visible region and narrow emission peak, colloidal and core/shell structured quantum dots (QDs) have been considered as one of the most efficient emitters for light‐emitting diodes (LEDs) which exhibit great application prospects in lighting and displays.^[^ ^1^, ^2^, ^3^, ^4^, ^5^ ^]^ In order to achieve high performance of QDs based LEDs (QLEDs), two important factors involving high PLQYs of QD materials and balanced charge carriers inside the light‐emitting devices are first considered.^[^ ^6^, ^7^, ^8^ ^]^ First of all, a prerequisite is to use the shell materials with certain thickness values, larger bandgaps, and fine lattice matching in comparison with the cores. It provides a perfect surface passivation and efficient confinement of charge carriers so as to result in high PLQYs of QDs.^[^ ^6^, ^7^, ^8^ ^]^ However, the generally used zinc sulfide (ZnS) shell only gives its QDs with an extremely deep valence band (VB = −6.9 eV) energy levels which significantly increase the energy barrier (>1.5 eV) for the hole injection from the hole transport layers (HTLs) to the emissive layers (EMLs) (**Figure** **1**a).^[^ ^8^, ^9^, ^10^, ^11^, ^12^ ^]^ Meanwhile, the electrons are transferred into the EMLs with a negligible obstacle owing to the fact that the energy levels of QDs are well matched with those of the highly conductive zinc oxide (ZnO) nanoparticles based electron transport layers (ETLs) (Figure 1a).^[^ ^13^, ^14^ ^]^ As a result, the recombination of holes and electrons inside the EMLs is unbalanced, significantly increasing the exciton quenching through the nonradiative channels and restricting the performance of QLEDs, such as high applied voltage, low efficiency, and significant efficiency roll‐off.^[^ ^15^, ^16^, ^17^ ^]^

To achieve much more balanced recombination, the general approaches would be to slow down the electron transport and decrease the energy barrier for hole injection at the interface of HTLs/EMLs.^[^ ^6^, ^18^, ^19^, ^20^, ^21^, ^22^ ^]^ For example, Peng and co‐workers reported an efficient red QLED by inserting an insulating interlayer PMMA (poly(methyl methacrylate)) between the QD EML and the ZnO ETL in 2014.^[^ ^6^ ^]^ The use of PMMA is to significantly slow down the electron transport and avoid the excitons inside the QD EML from being quenched by ZnO. As a result, the red QLED afforded a luminance of 42 000 cd m^−2^ at 8 V and exhibited a peak external quantum efficiency (EQE) of 20.5% at the luminance of around 1200 cd m^−2^. In this case, the distribution of charge carriers inside the EMLs is finely balanced. Howerver, the use of insulating interlayers results in a significant energy barrier for electron injection, leading to lower luminance values and higher applied votages at the threshold brightnesses (≈1000−10 000 cd m^−2^) for commercial applications. It is noted that the insulating film is usually ultrathin and not ready to reproduce.^[^ ^6^, ^23^ ^]^ On the other hand, by employing the selenium (Se) element throughout the whole core/shell regions, Shen and co‐workers designed a new type of QDs with the structures of CdSe/ZnCdSe/ZnSe, CdSe/ZnSe, and Zn_x_Cd_1‐x_Se/ZnSe for red, green, and blue (RGB) QDs, respectively. The devices made by these QDs exhibit high efficiencies at high luminance values. For example, the peak EQEs of RGB QLEDs are 21.6%, 22.9%, and 8.05% at the luminance values of 13 300, 52 500, and 10 100 cd m^−2^, respectively. The authors believe that the use of Se inside both the cores and shells results in a small VB offset with respect to TFB (poly (9,9‐dioctylfluorene‐*co*‐*N*‐(4‐(*sec*‐butyl)phenyl)diphenylamine)) HTL, which favours the hole injection and allows the balanced recombination of the holes and electrons inside the EMLs.^[^ ^7^ ^]^ Nevertheless, the energy barrier (≈1.3 eV) for hole injection from the HOMO of TFB to the VB of ZnSe shell is still large in these QLEDs and the challenge for hole injection in QLEDs is still not well resolved.

It is noted that most of the efficient QLEDs are achieved by using a typical type‐II QD structure, in which the bandgaps of the materials used in QDs are gradually increased from inner cores to outside shells.^[^ ^21^ ^]^ It creates a significant barrier for charge injection, especially for holes, although the use of shells with broad bandgaps is to achieve high PLQYs of QDs.^[^ ^19^ ^]^ Herein, we develop a new strategy in which CdZnS is used as an outermost shell material to achieve an unconventional type‐II QD structure, which is characterized by the initially increased but then decreased bandgaps from the inside cores to the outside shells (Figure 1b). It tunes the VBs of QDs to around −6.0 eV so as to well match with the HOMOs of HTLs (Figure 1b). As a result, the energy barrier from the HOMO of TFB to the VBs of QDs is decreased to be within 1.0 eV, which is in favour of the hole injection in the devices. We firstly used the CdZnS shell to synthesize the red QDs. By orderly coating ZnSe, ZnS, and CdZnS shells on the surface of CdZnSe core, the red QDs with a novel chemical structure of CdZnSe/ZnSe/ZnS/CdZnS (**R3**) were synthesized and investigated. On the one hand, CdZnS is generally used in the cores and interlayer shells, which is bound to afford a good lattice matching between the outside CdZnS and the other inside shell materials (such as ZnS and ZnSe).^[^ ^24^, ^25^, ^26^ ^]^ Therefore, high‐quality QDs with a perfect nanostructure are attained. On the other hand, the use of ZnS and ZnSe inner shells with a broad band gap is to confine the electrons and holes inside the core,^[^ ^14^ ^]^ affording **R3** with a strong luminescence at the emission wavelength of 620 nm and the PLQY of 94.4%. Most importantly, CdZnS possesses a shallower VB in comparison with ZnS and ZnSe, which could act as a springboard (energy barrier for hole injection less than 1.0 eV) to make the holes to be easily injected into the QD EMLs (Figure 1b), resulting in the balanced recombination of charge carriers in QLEDs.^[^ ^24^, ^25^, ^26^ ^]^ Therefore, the **R3** based red QLED (**D3**) exhibits an excellent performance with the peak EQE of over 20%, the luminance of 1.17 × 10^5^ cd m^−2^ at 6 V and operational lifetime (*T*_95_ at 100 cd m^−2^) of over 3.0 × 10^5^ h, significantly superior to its reference QLEDs made by the QDs with the outermost ZnSe and ZnS shells. A further application of the CdZnS shell material in the green and blue QLEDs was also investigated. The EQEs of the green and blue QLEDs fabricated by the CdZnS shell based QDs are 19.2% and 8.4%, respectively. In addition, these devices exhibit low applied voltages at the luminance values of 1000−10 000 cd m^−2^ and high power efficiencies. From these results, it is clear that the CdZnS material could serve as an efficient outermost shell and the hole injection springboard to afford highly efficient QLEDs.

## 2 Results and Discussion

### 2.1 Characterization of Red QDs

**Figure 2:**
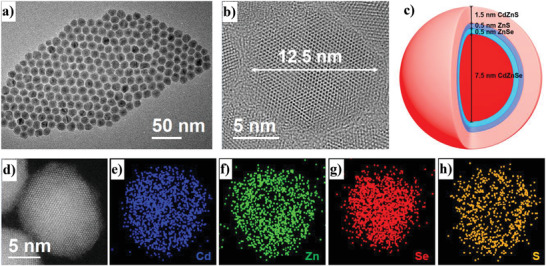
a,b) Transmission electron microscope (TEM) images of **R3** with different scale values; c) the corresponding core/shell structure of **R3**; d) high‐angle annular dark‐field scanning transmission electron microscopy (HAADF‐TEM) image of **R3**; e−h) energy dispersive spectroscopy elemental maps of Cd, Zn, Se, and S.

The CdZnS material was firstly used as the outermost shell of red QDs. These QDs with core/shell structures of CdZnSe/ZnSe (**R1**), CdZnSe/ZnSe/ZnS (**R2**), CdZnSe/ZnSe/ZnS/CdZnS (**R3**), CdZnSe/thick‐ZnSe (**R4**), and CdZnSe/ZnSe/thick‐ZnS (**R5**) (**Figure** **2**, Figures S1−S3, Supporting Information) were synthesized according to the processes shown in Supporting Information.^[^ ^27^, ^28^, ^29^, ^30^, ^31^ ^]^ The CdZnSe core was obtained by quickly injecting a Se solution into the mixture consisting of cadmium oleate and zinc oleate at a high temperature. Then, the prepared Se, S, and Cd precursors were injected into the core solution with different stoichiometric ratios to form the ZnSe (**R1** and **R4**), ZnS (**R2** and **R5**), and CdZnS (**R3**) shells and cover the surface of the CdZnSe core. **R1** and **R2** were used to investigate the thickness values of ZnSe, ZnS, and CdZnS shells of **R3**. Meanwhile, with comparable nanocrystal sizes and relatively thick ZnSe (or ZnS) shells, the reference **R4** and **R5** QDs can be used to study the influence of the CdZnS shell on the photophysics, VB energy levels, charge carrier conductivities, and device performances of **R3** after minimizing the size effect.

#### 2.1.1 Morphology and Structure Studies of Red QDs

It is noted that all the red QDs exhibit a sphere morphology due to their core/shell structures. **R3** exhibits a uniform size distribution with an average diameter of around 12.5 nm (Figure 2a,b and Figure S2d, Supporting Information), which is bigger than those of **R1** (≈8.5 nm), **R2** (≈9.5 nm), **R5** (≈10.5 nm), and the CdZnSe core (7.5 nm) (Figures S1 and S2, Supporting Information). The thickness values of ZnSe, ZnS, and outside CdZnS shells in **R3** are around 0.5, 0.5, and 1.5 nm, respectively (Figure 2c). Meanwhile, **R4** and **R5** possess the ZnSe and ZnS shells with the thickness values of around 2.75 and 1.5 nm, which are thicker than those of **R1** (0.5 nm) and **R2** (1.0 nm) (Figure S3, Supporting Information). From the image of high resolution transmission electron microscope (HR‐TEM), the surface of **R3** is partially shaded by organic ligands, resulting in a slightly obscure TEM image. Nevertheless, **R3** exhibits a perfect nanostructure with continuous lattice fringes and no lattice mismatch is observed (Figure 2b). In general, CdZnS is one of the core materials used in the blue and green QDs, which exhibits negligible lattice mismatch with respect to the ZnS and ZnSe shells.^[^ ^24^, ^25^, ^26^ ^]^ Also, it could be used as an intermediate shell to gradually change the chemical composition of QDs from the inner cores (or shells) to the outside shells, affording these QDs with less lattice mismatch and hence less interface defects.^[^ ^21^, ^32^ ^]^ Therefore, CdZnS could be used as the outermost shell material to get the red emitting CdZnSe/ZnSe/ZnS/CdZnS (**R3**) and afford **R3** with an excellent nanocrystal quality.^[^ ^33^ ^]^ In addition, the other red QDs also exhibit a fine core/shell structure and good nanocrystal quality. According to the thickness values of the core and shell in these QDs, the structures of QDs could be shown in Figure 2c and Figure S3 (Supporting Information). Approximately, the core/shell structures of **R1**−**R5** are CdZnSe (7.5 nm)/ZnSe (0.5 nm), CdZnSe (7.5 nm)/ZnSe (0.5 nm)/ZnS (0.5 nm), CdZnSe (7.5 nm)/ZnSe (0.5 nm)/ZnS (0.5 nm)/CdZnS (1.5 nm), CdZnSe (7.5 nm)/ZnSe (2.75 nm), and CdZnSe (7.5 nm)/ZnSe (0.5 nm)/ZnS (1.0 nm), respectively.

The chemical components in these QDs were also investigated by using the energy dispersive spectroscopy (EDS) and X‐ray photoelectron spectroscopy (XPS). To confirm that the outermost shell of **R3** consists of the Cd element, the HAADF‐STEM with an EDS element mapping was used to obtain the elemental maps of **R3**. A fine overlap between the distribution of elements (Cd and Zn) and the HAADF images in Figure 2e–h and Figure S4a (Supporting Information) demonstrates that the outermost shell of **R3** consists of Cd and Zn elements.^[^ ^34^ ^]^ By considering that the chemical reactivity of Cd is higher than that of Zn, Cd is firstly consumed and distributed inside the QDs.^[^ ^32^ ^]^ The formation of the outermost CdZnS shell in this work is due to the additional Cd precursor during the last shell growth rather than the diffusion of Cd from the inner to the outside, which could be consistent with other previous reports and confirmed by the element mapping of CdZnSe core.^[^ ^32^ ^]^ As shown in Figure S4b (Supporting Information), no distribution of Cd is found on the surface of the core and ZnSe is dominant in such an area, which reveals that the diffusion of Cd through the ZnSe layer to reach the surface is difficult in the absence of an additional force.^[^ ^35^ ^]^ Therefore, the Cd element mainly distributes inside the inner cores or intermediate shells of QDs in most cases.^[^ ^21^ ^]^ Meanwhile, the Zn 2p, Se 3d, S (or Se) 3p and Cd 3d peaks exhibit strong intensities in the XPS spectra, which reveals that Cd, Zn, S and Se are the main elements in these QDs (Figure S5, Supporting Information).^[^ ^36^ ^]^

#### 2.1.2 Photophysics of Red QDs

**Figure 3:**
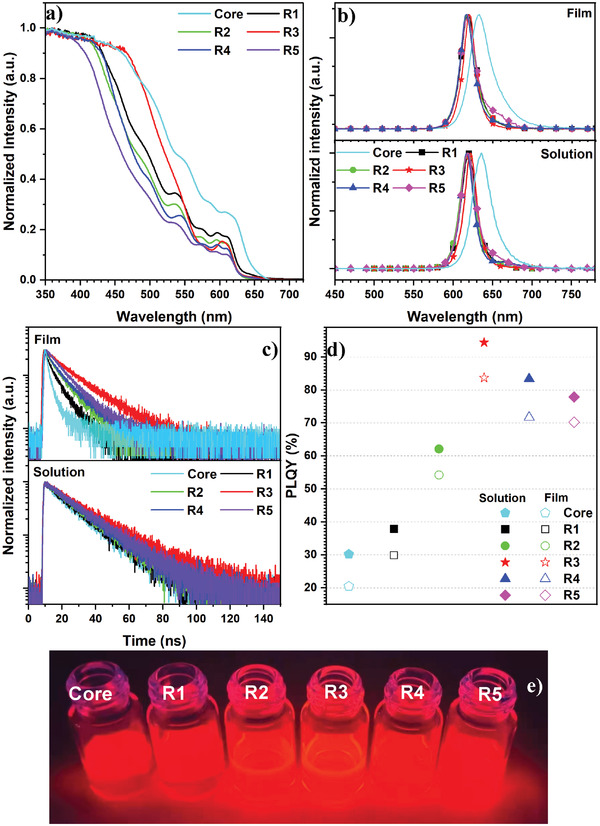
a) The UV–visible absorption spectra for the red QD solutions; b) the PL spectra of the red QD solutions and films; c) the PL decay curves; d) the PLQY values for the solutions and films of the red QDs; e) the images of red QD solutions under a 365 nm UV light.

By combining with the absorption and PL spectra (**Figure** **3**a,b), all the QDs exhibit a significant self‐absorption feature.^[^ ^24^, ^36^, ^37^, ^38^ ^]^ The use of 1.0 nm ZnS and ZnSe interlayer shells aims at providing a broad energy gap so as to well confine the recombination of electrons and holes inside the CdZnSe core, leading to an efficient light emission from the core instead of that from other shell materials. As shown in Figure 3b, the emission peaks of these QDs are at around 620 nm, confirming that the CdZnS outside the shell has a less influence on the peak emission wavelength of **R3**. The peak emission wavelengths for **R1**, **R2**, **R3**, **R4**, and **R5** are 618, 619, 622, 618, and 619 nm, respectively. The emission peaks are shifted from 636 nm of the CdZnSe core to around 620 nm of **R1**−**R5**. Once the shells were formed on the surface of core, the emission peaks of red QDs were all shifted toward the high‐energy region, which is similar to other previous reports.^[^ ^24^, ^36^, ^37^, ^38^ ^]^ It is mainly due to the diffusion of Zn from the interlayer into the CdZnSe core, slightly widening the energy gap of the core.^[^ ^25^ ^]^

In dilute solutions, these red QDs exhibit strong photoluminescence emissions with long decay lifetimes (*τ*). The single‐exponential decay curves of these QDs gave the *τ* values of 17.23, 17.38, 18.06, 22.28, 18.58, and 19.34 ns for the core and **R1**−**R5**, respectively (Figure 3c and Table S1, Supporting Information). The lifetimes of the red QDs increase with the thickness values of their shells, which reveals that the thicker shells afford the red QDs with a good surface passivation. The *τ* values measured from their films are 5.86, 5.98, 7.18, 13.61, 10.61, and 10.86 ns for the core and **R1**−**R5**, respectively (Figure 3c and Table S2, Supporting Information). Their longer *τ* values correspond to less interactions between each of the two QDs in their dilute solutions, restricting the nonradiative Förster resonant energy transfer (FRET). In comparison with the QD solutions, the packed QD films own relatively short *τ* values because of the enhanced FRET process after significantly increasing the interactions between two QDs.^[^ ^24^, ^39^ ^]^ It is noted that the **R3** film exhibits the single‐exponential decay, which demonstrates that a direct radiative recombination between the holes and electrons (that is, the intrinsic radiative decay) occurs. Besides, the biexponential decay curves for other QD films are observed, corresponding to two decay processes containing the intrinsic radiative decay and other processes (such as the trap of charge carriers by the surface defects of QDs). The PLQY of **R3** solution and thin film are 94.4% and 83.7%, respectively, which are significantly higher than those of others (core: 30.2% and 20.5%, **R1**: 37.9% and 29.9%, **R2**: 62.1% and 54.2%, **R4**: 83.4% and 71.7%, **R5**: 77.9% and 70.2%) (Figure 3d). In comparison with the QD solutions, the PLQY values of the QD films are generally decreased due to the FRET, which is consistent with the results of PL decay curves. There are mainly two reasons for the high PLQY of **R3**. On the one hand, the interlayer ZnSe and ZnS shells play an important role on confining the charge carriers and eliminating the lattice mismatch between the inner and outside shells. On the other hand, the outermost CdZnS shell on the surface of CdZnSe/ZnSe/ZnS QD further passivates the surface defects and affords **R3** with the perfect nanostructure. By combining with the longer lifetime and higher PLQY of **R3**, we believed that CdZnS could be used as the outermost shell and afford its QDs with high crystal qualities and hence excellent optical properties.

#### 2.1.3 The VB Studies of Red QDs

**Figure 4:**
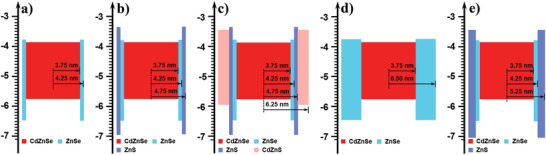
a−e) The VB energy levels of the core/shell structured red QDs (**R1**−**R5**).

To investigate the influence of the outermost CdZnS shell on the VB energy level of **R3**, the valence band spectra of these QDs were measured by ultraviolet photoelectron spectroscopy (UPS) with a monochromatic He I light source (21.21 eV) and a VG Scienta R4000 analyzer. The calculated VBs of the core and red QDs (**R1**−**R5**) are around −5.77, −6.49, −6.97, −5.97, −6.45, and −7.06 eV, respectively (Figure S6, Supporting Information). These VB energy levels of **R2**, **R4**, and **R5** are comparable to those of the previously reported ZnSe and ZnS shells based QDs.^[^ ^40^, ^41^ ^]^ It is noted that the outermost CdZnS shell could efficiently tune the VB energy level of **R3** to −5.97 eV, which is shallower than those of other ZnSe and ZnS shells based red QDs (**R1**, **R2**, **R4**, and **R5**). The probable VB and CB energy levels of QDs are shown in **Figure** **4**. The approximate CB values of ZnSe (−3.8 eV), ZnS (−3.4 eV), and CdZnS (−3.4 eV) are attained according to other literature reports because it is hard to accurately confirm these CB energy levels in our QDs.^[^ ^40^, ^42^ ^]^ Meanwhile, we put a focus on the influence of CdZnS shell on the energy barrier for hole injection from the HOMO of TFB to the VBs of QDs. In this case, the CB energy levels play a less influence on the hole injection. As shown in Figure 4c, the use of the outermost CdZnS shell is to arrange the energy levels of core and shell so that the energy level of **R3** is different to those of the generally reported core/shell structured QDs (such as **R1**, **R2**, **R4**, and **R5**). It affords **R3** with a new and unconventional energy level alignment. From the core to the outside shells, the bandgaps of ZnSe and ZnS are gradually increased and significantly broader than that of the CdZnSe core. Then, the energy gap of the outermost CdZnS is significantly narrower than those of the ZnSe and ZnS shells but slightly broader than that of the CdZnSe core. As a result, the outermost CdZnS shell with a shallow VB energy level could be used as the springboard (Figure 1b), which is in favor of hole injection from the HTLs into the QDs due to the significantly decreased energy barrier (0.64 eV) from the HOMO (−5.33 eV) of TFB to the VB (−5.97 eV) of **R3**.

### 2.2 QD Based Devices

#### 2.2.1 Single Charge Carrier Devices

To confirm that the outermost CdZnS shell with a relatively shallow VB energy level could be used as the hole injection springboard, the hole‐only devices (HODs) were fabricated by using the device structure of indium tin oxide (ITO)/poly(3,4‐ethylenedioxythiophene):poly(styrenesulfonate) (PEDOT:PSS, 40 nm)/TFB (30 nm)/QDs (10−20 nm)/molybdenum oxide (MoO_3_, 3 nm)/aluminum (Al, 100 nm). From the current density−voltage (*J*−*V*) curves (Figure S7a, Supporting Information), it is reasonable that the HODs made by **R1** and **R2** own larger *J* values than those of HODs made by other QDs due to their small diameters. Here, we only compare the hole conductivities among the HODs fabricated by **R3**, **R4**, and **R5** because the comparable thickness values of their shells minimize the influence of size on the hole conductivity and only the energy gaps from the HOMO of TFB to the VBs of QDs are involved. It is noted that the HOD based on **R3** transports much more holes through the device in comparison with the HODs based on **R4** and **R5**, corresponding to a better hole conductivity of **R3** than those of **R4** and **R5** (Figure S7a, Supporting Information).^[^ ^20^, ^22^ ^]^ Therefore, the outermost CdZnS shell with the VB of −5.97 eV can be used as the hole injection springboard and helps more holes to be injected into the QD EMLs (Figure 1b,c). On the other hand, the electron‐only devices (EODs) with the device structures of ITO/magnesium doped ZnO (ZnMgO, 40 nm)/QDs (15−20 nm)/ZnMgO (40 nm)/Al (100 nm) were also fabricated. Relatively high current densities of the EODs based on **R1**, **R2**, **R4**, and **R5** were observed from their *J*−*V* curves (Figure S7b, Supporting Information). It is mainly due to the small diameters of **R1** and **R2**, and nearly 100% ZnSe and ZnS proportions in the shells of **R4** and **R5**. Most importantly, the well matched current densities of electrons and holes were observed in the HOD and EOD based on **R3** (Figure S7c, Supporting Information), corresponding to much more balanced holes and electrons inside **R3**. However, the current densities of these HODs and EODs made by other QDs exhibit a much more significant difference, corresponding to an unbalanced distribution of charge carriers inside the EMLs. As a result, the outermost CdZnS shell could act as the springboard for hole injection, affording the devices based on **R3** with much more balanced charge carriers.

#### 2.2.2 Device Structure and Performance of the Red QLEDs

**Figure 5:**
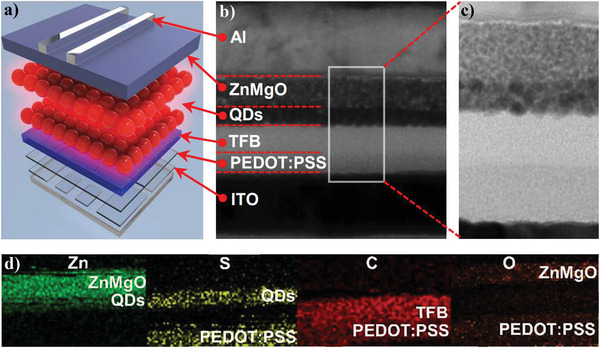
a) Device structure of **D3**; b,c) cross‐sectional TEM images of **D3**; d) EDS compositional mapping images of the corresponding section in (c).

As shown in **Figure** **5**a,b, the red QLEDs were fabricated by orderly stacking the PEDOT:PSS (40 nm) HIL, TFB HTL (30 nm), QD EMLs (15−20 nm), and ZnMgO ETL (40 nm) on the surface of ITO anode through solution‐process. Then 100 nm Al was deposited on the top of ZnMgO as the cathode by thermal evaporation (see the Supporting Information).^[^ ^43^ ^]^ From the image of cross‐sectional TEM, clear boundaries at the interfaces of EML/ETL and EML/HTL were observed (Figure 5b,c). It is noted that the thickness of **R3** is around 20 nm, corresponding to 1.5−2.0 QD layers in **D3**. The elements of Zn, S, C, and O are found in these compositional mapping images measured by EDS, corresponding to these functional layers in **D3** (Figure 5d).

**Figure 6:**
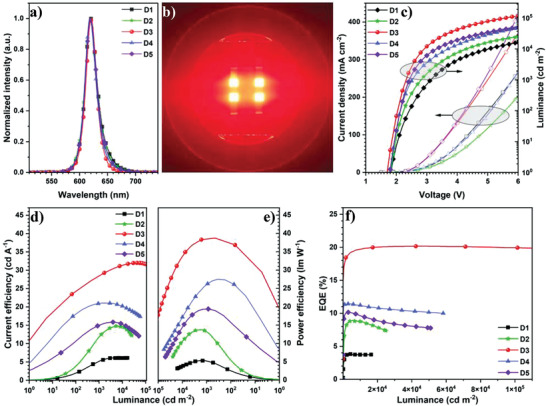
a) EL spectra of red QLEDs; b) the image of **D3**; c) *J*−*V*−*L* curves; d) CE, e) PE, and f) EQE−*L* curves of the devices.

The red QLEDs fabricated by using **R1** (**D1**), **R2** (**D2**), **R3** (**D3**), **R4** (**D4**), and **R5** (**D5**) were measured by an EQE measurement system (C9920‐12, Hamamatsu Photonics, Japan) containing an integrating sphere and multi‐channel analyzer PMA‐12.^[^ ^44^ ^]^ They exhibit red emissions at the emission wavelengths of around 620 nm with the full width at half maximum (FWHM) values of around 25 nm (**Figure** **6**a). Due to a better nanostructure and small size distribution, the electroluminescence (EL) spectrum of **D3** exhibits a relatively small FWHM value of 22 nm, which is smaller than **D1** (25 nm), **D2** (26 nm), **D4** (24 nm), and **D5** (25 nm) (Figure 6a). The Commission Internationale de l'Eclairage (CIE) chromaticity coordinates of these red QLEDs are (0.67, 0.32), (0.67, 0.32), (0.68, 0.32), (0.67, 0.33), and (0.68, 0.32) for **D1**−**D5** (**Table** **1**), respectively, corresponding to the pure red region (Figure 6b). Due to the decreased energy barrier from the HOMO (−5.33 eV) of TFB to the VB (−5.97 eV) of the CdZnS shell (Figure S8a, Supporting Information), the holes are easily and efficiently injected into the EML of **D3**, affording **D3** with a lower turn‐on voltage (*V*_on_ = 1.7 V) and higher luminance (*L* = 1.17 × 10^5^ cd m^−2^) at the applied voltage of 6 V (Figure 6c). The luminance of **D3** can be over 4.0 × 10^5^ cd m^−2^ when the applied voltage is increased to 15 V (Figure S8b,c, Supporting Information), which is among the brightest red QLEDs.^[^ ^45^ ^]^ Therefore, **D3** exhibits relatively higher efficiencies than other devices. For example, the peak current and power efficiencies (CE and PE) for **D3** are 32.0 cd A^−1^ and 38.8 lm W^−1^, respectively, significantly superior to those of **D1** (6.2 cd A^−1^ and 5.3 lm W^−1^), **D2** (14.8 cd A^−1^ and 13.7 lm W^−1^), **D4** (21.1 cd A^−1^ and 27.5 lm W^−1^), and **D5** (15.9 cd A^−1^ and 19.4 lm W^−1^) (Figure 6d,e and Table 1). It is noted that the peak EQE of 20.2% for **D3** is significantly superior to those of **D1** (3.8%), **D2** (8.9%), **D4** (11.5%), and **D5** (10.2%) (Figure 6f and Table 1). Meanwhile, when the brightness is in the region from 3.0 × 10^4^ to 9.0 × 10^4^ cd m^−2^, the EQE value of over 20.0% is observed (Figure 6f). It is noted that such small EQE drop at high brightness corresponds to a drop‐free red QLED.^[^ ^45^ ^]^ Besides, **D3** exhibits a good EQE reproducibility and operational stability (Figure S9, Supporting Information). When the initial luminance is 5000 cd m^−2^, **D3** owns a longer operational lifetime (*T*_95_ = 265.3 h) than other reference devices, corresponding to the *T*_95_ value of over 3.0 × 10^5^ h when the luminance is 100 cd m^−2^ (Table 1 and Figure S9, Supporting Information).

**Table 1:** The electroluminescence performance of QLEDs

QLEDs	*λ*_EL_ [nm]	FWHM [nm]	CIE [*x*, *y*]	*V*_on_ [V]	*L* at 6 V [cd m^−2^]	EQE [%]	PE [lm W^−1^]	CE [cd A^−1^]	*T* [h]
**D1**	619	25	(0.67,0.32)	1.9	16300	3.8	5.3	6.2	2972^a)^
**D2**	618	26	(0.67,0.32)	1.9	24892	8.9	13.7	14.8	100037^a)^
**D3**	620	22	(0.68,0.32)	1.7	117000	20.2	38.8	32.0	303313^a)^
**D4**	618	24	(0.67,0.33)	1.8	58269	11.5	27.5	21.1	252208^a)^
**D5**	619	25	(0.68,0.32)	1.8	50856	10.2	19.4	15.9	122331^a)^
**D6**	535	23	(0.21,0.75)	1.9	137000	19.2	103.5	84.9	66554^b)^
**D7**	476	24	(0.11,0.13)	2.4	12000	8.4	8.3	7.7	10.6^b)^

^a)^ *T*_95_ values for the operational lifetimes of devices;

^b)^ *T*_50_ values for the operational lifetimes of devices.

We believe that the excellent performance of **D3** is mainly ascribed to the reasons listed below. On the one hand, **R3** with the high‐quality nanostructure possesses a higher PLQY than those of other QDs. On the other hand, the use of CdZnS hole injection springboard significantly decreases the energy gap for hole injection from the HOMO of TFB to the VB of **R3** in **D3** to 0.64 eV (Figure S8a, Supporting Information). To study the reason for the improved performance of **D3**, electrochemical impedance spectroscopy (EIS) was used to further investigate the physical process of these red QLEDs. The Nyquist plots of EIS spectra were measured at the turn‐on voltages of red QLEDs, and they were fitted according to the equivalent circuit (Figure S10, Supporting Information), which was used in other reports.^[^ ^20^, ^22^ ^]^ In the equivalent circuit, *R*_s_, *R*_tr_, and *R*_rec_ represent the overall external resistance (which is generated from electrodes, connected wires and interfaces between the electrodes and functional layers), charge transfer based resistance and recombination resistance, respectively. Meanwhile, CPE_1_ and CPE_2_ correspond to the interfacial capacitance of charge transfer layer and the chemical capacitance generated from the recombination area, respectively. As we know, strong conductivity of electrons in ZnMgO and no barrier for electron injection from ZnMgO ETLs to QD EMLs drive the electrons to be overinjected in most of the QLEDs. Therefore, the *R*_tr_ values are mainly related to the hole injection from the TFB HTL to the QD EMLs in this work since the differences of these QLEDs lie only in the QD materials used. It is found that the *R*_tr_ values are increased with the thickness of shell and barrier for hole injection (Table S3, Supporting Information). Although **R3** owns a much thicker shell among these red QDs, it still exhibits the lowest *R*_tr_, which corresponds to the best hole injection from TFB to **R3** in **D3** mainly due to the decreased energy barrier between the HOMO of TFB and VB of **R3**. Moreover, the *R*_rec_ values are negatively correlated to the recombination rates in QLEDs. It is noted that **D3** gives the smallest *R*_rec_ value (Table S3, Supporting Information). In other words, **D3** exhibits the most efficient recombination of charge carriers among these QLEDs, which is consistent with the highest PLQY of **R3**.^[^ ^20^, ^22^ ^]^ Therefore, the high‐quality and efficiently emissive **R3** along with its feature of favorable hole injection in the QLED lead to high‐brightness, high‐efficiency and good stability of **D3**.

#### 2.2.3 Performance of Green and Blue QLEDs

**Figure 7:**
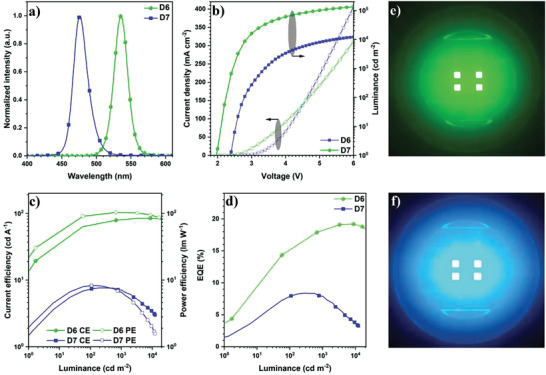
The performance of the green and blue QLEDs fabricated by the CdZnS shell based QDs: a) EL spectra; b) *J*−*V*−*L* curves; c) CE/PE−*L* curves; d) EQE−*L* curves; e,f) the images of working green and blue QLEDs.

The outermost CdZnS shell was also used to synthesize the green and blue QDs by using the structures of CdZnSe/ZnSe/ZnSeS/CdZnS (**GQD**) and CdZnSe/ZnSeS/ZnS/CdZnS (**BQD**), respectively (Supporting Information). In comparison with **R3**, the change is used to tune the peak emission wavelengths of these QDs into the high‐energy emission region. As shown in Figures S1f,g (Supporting Information), **GQD** and **BQD** also exhibit high‐quality nanocrystals with the size values of 11.0 and 7.0 nm, respectively. The optical properties of **GQD** and **BQD** are shown in Figure S11, Tables S1 and S2 (Supporting Information). From the PL tests, the peak emission wavelengths of **GQD** and **BQD** are located at 537 and 477 nm with the PLQYs of 93.8% and 73.3%, respectively (Figure S11b,d, Supporting Information). Meanwhile, the UPS results reveal that the VB energy levels of **GQD** and **BQD** are around −6.07 and −6.09 eV (Figure S6g,h, Supporting Information), resulting in the energy gaps of around 0.74 and 0.76 eV from the HOMO of TFB to the VBs of QDs. Therefore, the energy barriers for hole injection in these QLEDs are still smaller than 1.0 eV, which is in favor of hole injection into the QD EMLs. The QLEDs based on **GQD** (**D6**) and **BQD** (**D7**) possess low *V*_on_ values of 1.9 and 2.4 V and high luminance values of over 1.37 × 10^5^ and 1.2 × 10^4^ cd m^−2^ at 6 V (**Figure** **7**b). The low turn‐on voltages and high luminance values correspond to an easy hole injection into the QD EMLs arising from the use of the CdZnS shell. The EQEs of **D6** and **D7** are 19.2% and 8.4%, respectively (Figure 7d). It is noted that the PE of **D6** is 103.5 lm W^−1^, which is among the highest PE values for green QLEDs (Figure 7c).^[^ ^46^ ^]^ These devices exhibit the peak emission wavelengths of 535 and 476 nm with the FWHM values of 23 and 24 nm (Figure 7a), corresponding to the CIE coordinates of (0.21, 0.75) and (0.11, 0.13) for **D6** and **D7**, respectively (Figure 7e,f and Table 1). Besides, the green and blue QLEDs also possess high EQE repeatabilities with operational lifetimes (*T*_50_) of 146.0 and 10.6 h for **D6** and **D7**, respectively (Figure S12, Supporting Information). The performance of the blue QLED (**D7**) is inferior to the red (**D3**) and green (**D6**) QLEDs, which is mainly due to the fact that the smaller nanocrystal size of **BQD** results in much more surface defects and hence a lower PLQY (Figures S1g and S11d, Supporting Information). Further research works to improve the quality of the blue QD based on CdZnS shell are now in progress.

## 3 Conclusion

Herein, the outermost CdZnS shell based RGB QDs were designed and synthesized. The core/shell structures are CdZnSe/ZnSe/ZnS/CdZnS, CdZnSe/ZnSe/ZnSeS/CdZnS, and CdZnSe/ZnSeS/ZnS/CdZnS for RGB QDs, respectively. CdZnS is selected as the outermost shell according to the reasons listed below. On the one hand, the less lattice mismatch between the inner ZnS (or ZnSe) and the CdZnS shells affords these new QDs with high nanocrystal qualities and excellent PLQYs of 94.4%, 93.8%, and 73.3% for RGB QDs, respectively. On the other hand, it could be used as a hole injection springboard for its shallow VB energy level. Therefore, the QLEDs based on these new QDs exhibit much more balanced charge carriers inside their QD EMLs and hence an excellent performance, affording the RGB light‐emitting devices with the peak EQEs of 20.2%, 19.2%, and 8.4%, respectively. Among these devices, the red QLED (**D3**) exhibits the features of high luminance (over 4.0 × 10^5^ cd m^−2^), well‐restricted EQE roll‐off (drop‐free at the luminance of 9.0 × 10^4^ cd m^−2^) and long operation stability (*T*_95_ of 3.0 × 10^5^ h). Meanwhile, the green QLED (**D6**) owned a high PE of over 100 lm W^−1^. From these results, CdZnS can be used as the outermost shell to achieve highly luminescent RGB QDs and act as the hole injection springboard so as to balance the charge carriers inside their QLEDs.

## Conflict of Interest

The authors declare no conflict of interest.

## Supporting information

Supporting Information

## Data Availability

The data that support the findings of this study are available from the corresponding author upon reasonable request.
