# Supplementary material for: Cadmium‐Doped Zinc Sulfide Shell as a Hole Injection Springboard for Red, Green, and Blue Quantum Dot Light‐Emitting Diodes
Source: Adv Sci (Weinh). 2022 Mar 3;9(15):2104488. doi: 10.1002/advs.202104488 (PMC9131609; doi:10.1002/advs.202104488)
Supplement: Supplementary file 1 — Supporting Information [file ADVS-9-2104488-s001.pdf]

ADVANCED SCIENCE
Open Access

Supporting Information

for *Adv. Sci.*, DOI 10.1002/advs.202104488

Cadmium-Doped Zinc Sulfide Shell as a Hole Injection Springboard for Red, Green, and Blue Quantum Dot Light-Emitting Diodes

Bochen Liu, Yue Guo, Qiang Su, Yunfeng Zhan, Zhao Chen, Yang Li, Baogui You, Xiaonan Dong, Shuming Chen* and Wai-Yeung Wong**

Supporting Information

Cadmium-Doped Zinc Sulfide Shell as a Hole Injection Springboard for Red, Green and Blue Quantum Dot Light-Emitting Diodes

Bochen Liu, Yue Guo, Qiang Su, Yunfeng Zhan, Zhao Chen*, Yang Li, Baogui You, Xiaonan Dong, Shuming Chen* and Wai-Yeung Wong*

B. Liu, Dr. Y. Guo, Dr. Y. Zhan and Dr. Z. Chen
School of Applied Physics and Materials, Wuyi University, Jiangmen, 529020, P. R. China
E-mail: chenzhao2006@163.com
Q. Su and Dr. S. Chen
Department of Electrical and Electronic Engineering, Southern University of Science and Technology, Shenzhen, 518055, P. R. China
E-mail: chen.sm@sustc.edu.cn
Prof. W.-Y. Wong
Department of Applied Biology and Chemical Technology, Research Institute for Smart Energy and Guangdong-Hong Kong-Macao Joint Laboratory for Photonic-Thermal-Electrical Energy Materials and Devices, The Hong Kong Polytechnic University (PolyU), Hung Hom, Hong Kong, P. R. China
E-mail: wai-yeung.wong@polyu.edu.hk
Prof. W.-Y. Wong
PolyU Shenzhen Research Institute, Shenzhen 518057, P. R. China.
Dr. Y. Li, Dr. B. You and X. Dong
Poly Optoelectronics Tech. Ltd, Jingmen, 529020, P. R. China
Dr. Y. Li and Dr. B. You
Fujian Science & Technology Innovation Laboratory for Optoelectronic Information of China, Fuzhou City, 350108, P. R. China

B.L. and Y.G. contributed equally to this work.

Synthesis of QDs

Precursor preparation:[1] A 2 M Se-TBP (tributyl phosphine) precursor was prepared by adding 10 mmol Se powder (780 mg) and 5 mL TBP solution into a 25 mL reaction tube. The mixture was heated under an inert environment to obtain a transparent Se-TBP solution. For CdZnS shell growth, a Cd precursor (0.2 M cadmium oleate, $Cd(OA)_2$) was synthesized by dissolving 2 mmol CdO (256 mg) into a 50 mL two-necked flask containing 10 mL oleic acid (OA) solution. The mixture was degassed at 135 ^{o}C for 1 h. Then it was heated to 280 ^{o}C under nitrogen for another hour to obtain the Cd precursor. Lastly, a S-TBP precursor was prepared according to the route similar to the Se-TBP precursor. All these precursors were preserved under nitrogen and directly used unless heating is needed.

Red emitting CdZnSe core:[2] CdO (64 mg, 0.5 mmol), ZnO (1628 mg, 20 mmol), OA (20 mL) and 1-octadecene (ODE, 30 mL) were placed into a 100 mL three-necked flask. The mixture was heated to 80 °C to form a transparent solution under a reduced pressure and then degassed at 135 ^{o}C for 1 h. With the protection of nitrogen, the reaction temperature was quickly elevated to 300 ^{o}C. 1 mL 2 M Se-TBP solution was quickly injected into the flask to form CdZnSe core at 300 ^{o}C and the mixture was kept at such a temperature for 1 h for efficient core growth. The CdZnSe core could be directly used to synthesize the red QDs without any purification. To characterize this core, this mixture was washed with a mixture of octane and ethanol when the reaction temperature was decreased to room temperature. Then, the precipitate was collected by centrifugation at the speed of 6 000 rpm and redispersed into octane to prepare the QD solution (30 mg mL^{-1}).

CdZnSe/ZnSe (**R1**):[3–5] The first layer ZnSe shell was grown on the surface of CdZnSe core by injecting 0.5 mL 2M Se-TBP precursor into the reactor at 300 ^{o}C for 1 h. Like the core, **R1** could be directly used for the shell growth or purified for the morphology and photophysics studies.

CdZnSe/ZnSe/ZnS (**R2**): To form the second ZnS shell, 1-dodecanethiol (DDT) was slowly added into the flask containing **R1** at 300 ^{o}C for 1 h.

CdZnSe/ZnSe/ZnS/CdZnS QDs (**R3**): For the third CdZnS shell, 3 mL 0.2 M $Cd(OA)_2$ solution and 0.6 mL 1 M S-TBP were swiftly injected into the flask consisting of **R2** and the mixture was kept at 300 ^{o}C for 1 h. Then, the obtained CdZnSe/ZnSe/ZnS/CdZnS QDs (**R3**) were purified according to the method used to purify the CdZnSe core.

CdZnSe/thick-ZnSe QDs (**R4**): **R4** was obtained by slowly injecting 1.5 mL Se-TBP (2 M) precursor into the **R1** solution at 300 ^{o}C within 1.5 h.

CdZnSe/ZnSe/thick-ZnS QDs (**R5**): **R5** was synthesized via covering the **R2** by using the ZnS shell. 0.2 mL S-TBP (1 M) precursor was injected into **R2** and the mixture was kept at 300 ^{o}C for 1 h. These QD solutions (30 mg mL^{-1}) were stored into a refrigerator with a temperature of around 5 ^{o}C before use.

CdZnSe/ZnSe/ZnSeS/CdZnS (**GQD**): The synthesis route was slightly adjusted to obtain the green emissive **GQD**. ZnO (1628 mg, 20 mmol), OA (20 mL) and ODE (30 mL) were placed into a 100 ml three-necked flask. The mixture was heated to 80 °C to form a transparent solution under a reduced pressure and then degassed at 135 ^{o}C for 1 hour. The reaction temperature was quickly elevated to 250 ^{o}C under nitrogen. 3 mL 2 M Se-TBP solution was quickly injected into the flask and the temperature was decreased to 150 ^{o}C. At such a temperature, 9 mL 0.2 M $Cd(OA)_2$ was quickly injected and the temperature was quickly elevated to 310 ^{o}C for 1.5 hour. After the CdZnSe core formation, the temperature was controlled at 300 ^{o}C and then 2 mL 2 M Se-TBP was added into the mixture for 1 hour for the ZnSe shell growth. For the ZnSeS shell, 1 mL Se-TBP and 2 mL 1 M S-TBP were added into the reactor together for 1 hour. The outside CdZnS shell was formed on the ZnSeS shell by adding 1 mL S-TBP and 5 mL 0.2 M $Cd(OA)_2$ at 300 ^{o}C for 1 hour. The **GQD** was purified according to the approach used before.

CdZnSe/ZnSeS/ZnS/CdZnS (**BQD**): ZnO (1628 mg, 20 mmol), OA (20 mL) and ODE (30 mL) were placed into a 100 ml three-necked flask. The mixture was heated to 80 °C to form a transparent solution under a reduced pressure and then degassed at 135 ^{o}C for 1 hour. The reaction temperature was quickly elevated to 270 ^{o}C under nitrogen. 3 mL 2 M Se-TBP solution was quickly injected into the flask. When the

temperature was increased to 310 ^{o}C, 4 mL 0.2 M $Cd(OA)_2$ was rapidly injected and the temperature was kept at 310 ^{o}C for 2 hour to form the CdZnSe core. The first ZnSeS shell was formed through injecting 0.5 mL Se-TBP and 1 mL S-TBP at the temperature of 310 ^{o}C for 1 hour. Then 0.5 mL S-TBP was added into the mixture for another 1 hour. When the temperature was decreased to 280 ^{o}C, 0.5 mL S-TBP and 0.2 mL $Cd(OA)_2$ were added into the reactor and it was heated for 1 hour. The **BQD** was purified according to the approach used before.

Optical and structural charaterization of QDs

These QDs were fully characterized by an UV-visible absorption spectroscopy, steady- and transient-state photoluminescence (PL) spectroscopy, transmission electron microscopy (TEM), X-ray photoelectron spectroscopy (XPS) and ultraviolet photoelectron spectroscopy (UPS). The UV-visible spectra were measured on an UV/VIS/NIR spectrometer Lambda 950 (PerkinElmer). For the solution samples (2.0 mg mL^{-1}), a quartz cuvette with the length of 1 cm was used as the holder. The PL spectra and PL decay curves were recorded from an Edinburgh Instruments Spectrometer FSL980. A xenon lamp and light-emitting diode with the excitation wavelength of 375 nm were selected as the light sources. The absolute PLQYs were measured using the Quantaurus-QY Plus C13534-12 (Hamamatsu, Japan). TEM images were obtained using a JEM-2100HR or JEM-3100F microscope. XPS was recorded on a Thermo-ESCA-Lab 250 instrument. The UPS spectra were measured by using a monochromatic He I light source (21.22 eV) and a VG Scienta R4000 analyzer.

Fabrication, structure and measurement of devices

Device fabrication and structures: The indium tin oxide (ITO) substrates with a sheet resistance of around 35 Ω were cleaned in an ultrasonic cleaner by orderly using detergent and deionized water. Firstly, for the hole-only devices, the ITO substrates were treated with UV-O_3 for 45 min. Then, 40 nm poly(3,4-ethylenedioxythiophene):poly(styrenesulfonate) (PEDOT:PSS, HIL) was

fabricated on the ITO anode by spin-coating and placed on a hot plate (150 °C) for 15 min in air. The substrates were transferred into a nitrogen filled glove box. The hole transporting polymer poly(9,9-dioctylfluorene-*co*-*N*-(4-(*sec*-butyl)phenyl)diphenylamine) (TFB) with a concentration of 8 mg mL^{-1} in chlorobenzene was spin-coated on the HIL and annealed at 120 °C for 10 min to obtain a 30 nm HTL. The QD films (15−20 nm) were fabricated by spin-coating the QD solutions (15 mg mL^{-1} in octane) on the HTL and annealed at 90 °C for 5 min. Under a pressure of less than 2×10^{-6} torr, 3 nm molybdenum oxide (MoO_3) and 100 nm aluminium (Al) cathode were thermally deposited. Therefore, the device architectures of hole-only devices are ITO/PEDOT:PSS (40 nm)/TFB (30 nm)/QDs (15−20 nm)/MoO_3 (3 nm)/Al (100 nm). Secondly, the electron-only devices were fabricated by using the device structures of ITO/magnesium doped ZnO (ZnMgO, 40 nm)/QDs (15−20 nm)/ZnMgO (40 nm)/Al (100 nm). The ZnMgO (ETL), QD and ZnMgO films were fabricated on the ITO subtrates in sequence by spin-coating in an inert atmosphere. Lastly, the RGB QLEDs were fabricated by spin-coating or thermally depositing the HIL, TFB HTL, QD EMLs, ZnMgO ETL and Al cathode on the surface of UV-O_3 pre-cleaned ITO substrtes to construct the architectures of ITO/PEDOT:PSS (40 nm)/TFB (30 nm)/QDs (15−20 nm)/ZnMgO (40 nm)/Al (100 nm).[6] These QLEDs based on **R1**−**R5**, green (**GQD**) and blue QDs (**BQD**) were denoted by device **D1**−**D7**.

Device characteristics and measurements: Particularly, the cross-sectional TEM of **D3** was investigated by using field emission transmission electron microscope and energy dispersive spectroscopy (EDS) (JEM-3100F). The devices with an active area of 2 mm × 2 mm were encapsulated before measurement. The current−voltage (*I*−*V*) curves were measured by a dual-channel Keithley 2400 source meter. For the charge-only devices, these *I*−*V* curves were measured in a dark box. For the QLEDs, an external quantum efficiency measurement system (C9920−12, Hamamatsu Photonics, Japan) containing an integrating sphere and multi-channel analyzer PMA−12 was used to collect the EL spectra, luminance values and EQEs.[7]

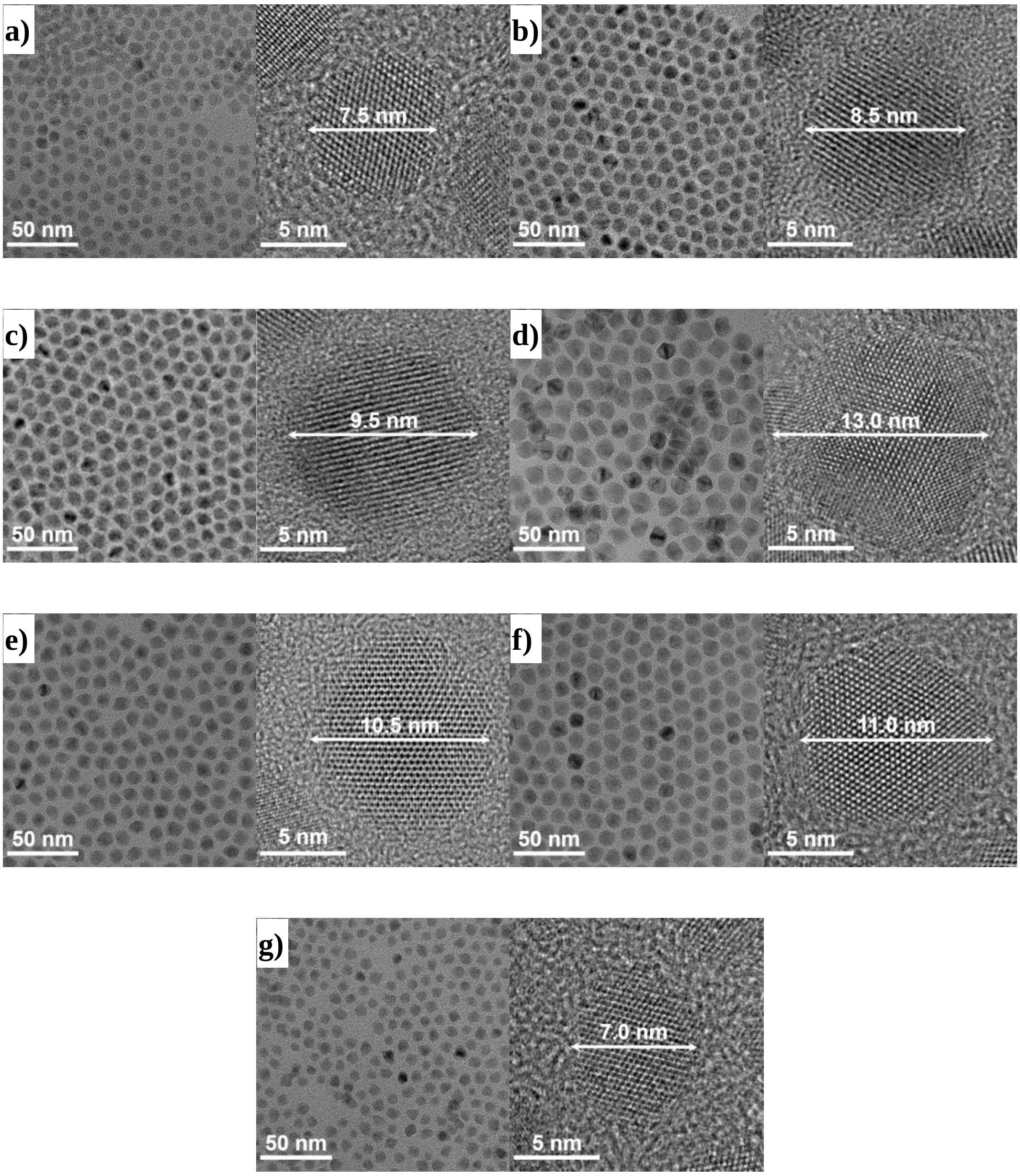

Figure S1. The TEM images of QDs: a) core, b) **R1**, c) **R2**, d) **R4**, e) **R5**, f) **GQD** and g) **BQD**.

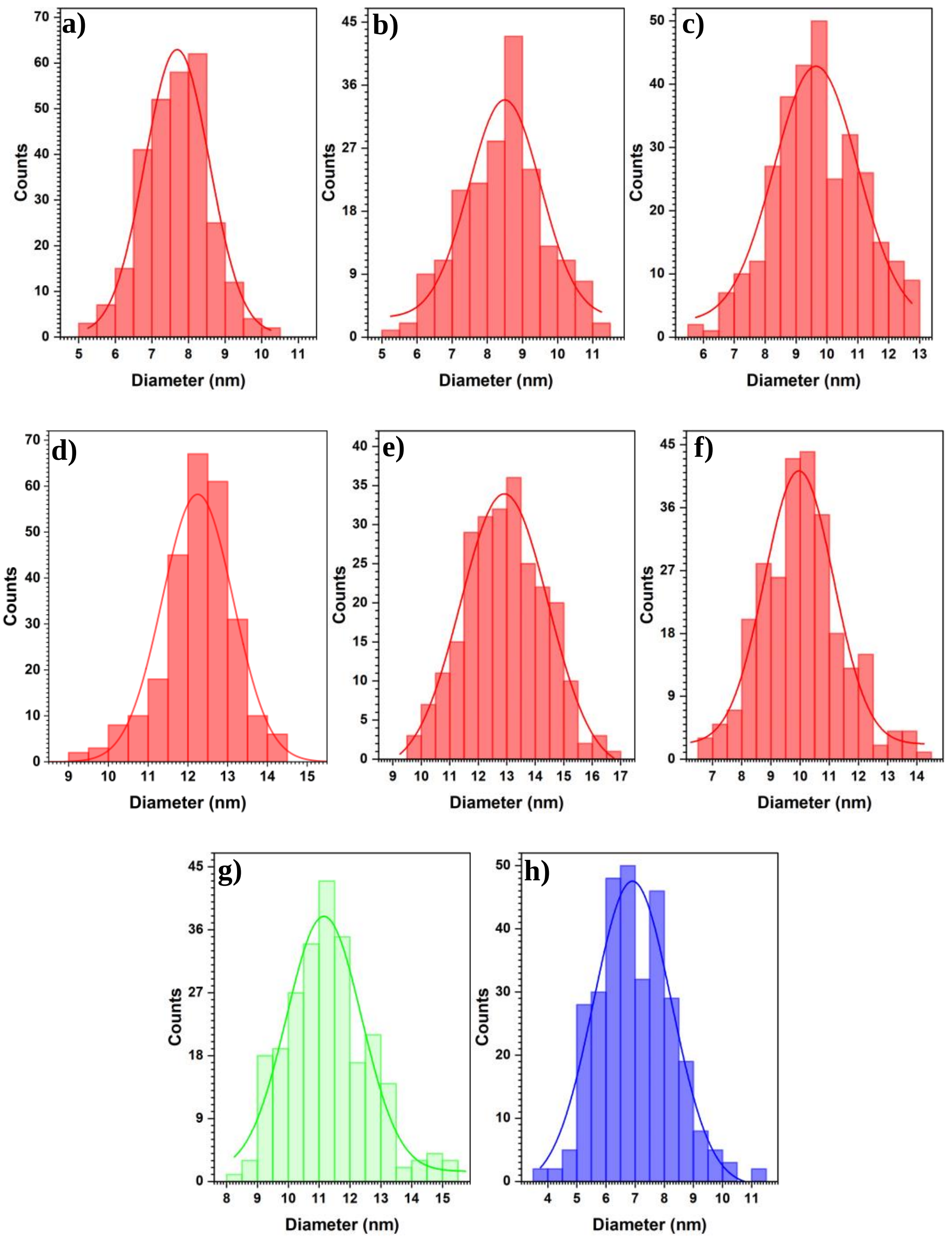

Figure S2. The size distribution of a) core, b) **R1**, c) **R2**, d) **R3**, e) **R4**, f) **R5**, g) **GQD** and h) **BQD**.

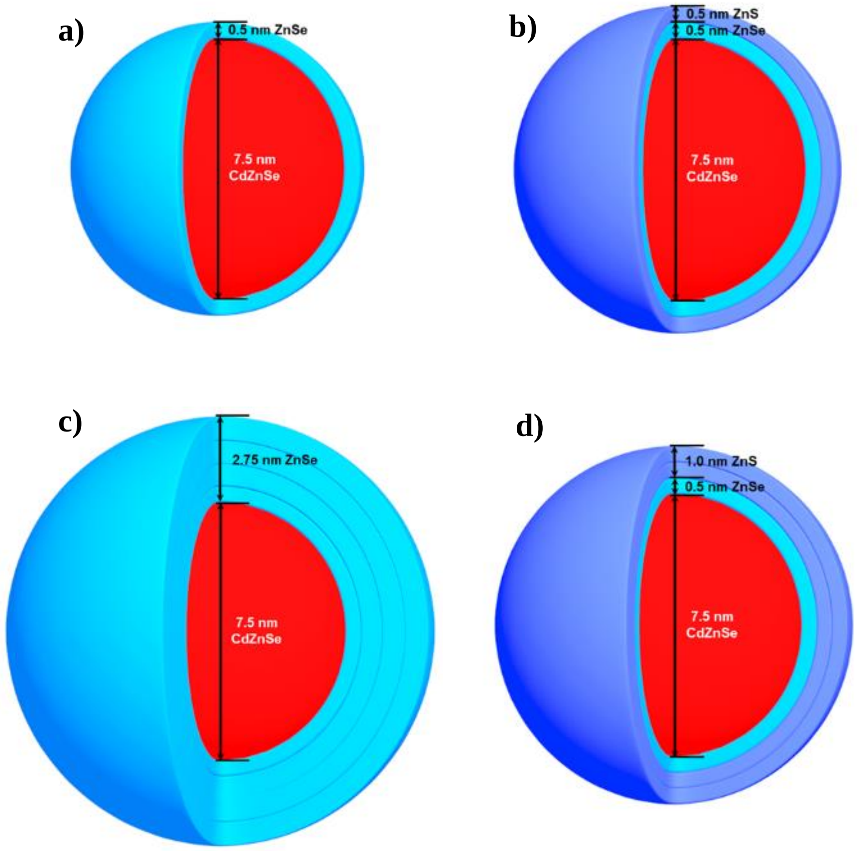

Figure S3. The core/shell structures of a) **R1**, b) **R2**, c) **R4** and d) **R5**.

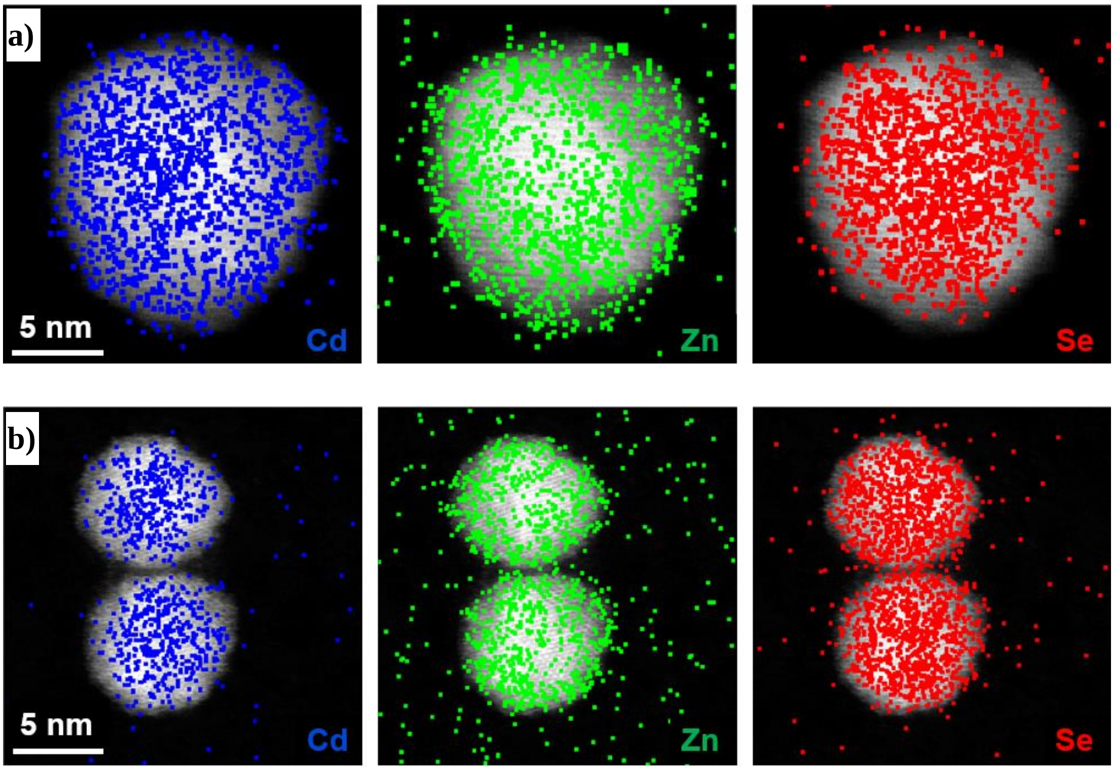

Figure S4. The distribution of Cd, Zn and Se elements in a) **R3** and b) CdZnSe core.

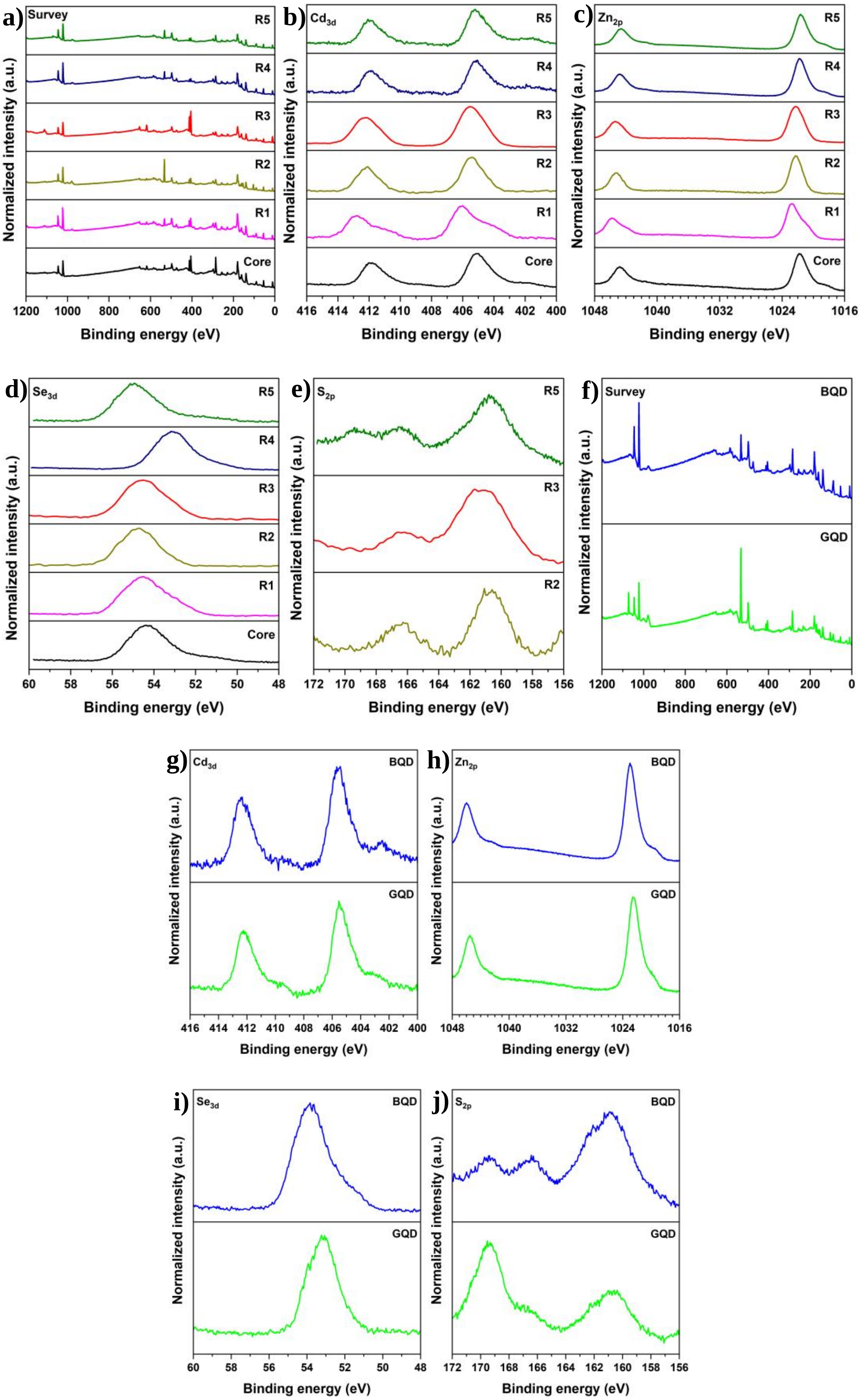
a) Survey
b) Cd3d
c) Zn2p
d) Se3d
e) S2p
f) Survey
g) Cd3d
h) Zn2p
i) Se3d
j) S2p
R5
R4
R3
R2
R1
Core
BQD
GQD
Normalized intensity (a.u.)
Binding energy (eV)

Figure S5. The XPS spectra of QDs: a)−e) for core, **R1**−**R5**; f)−j) for **GQD** and **BQD**.

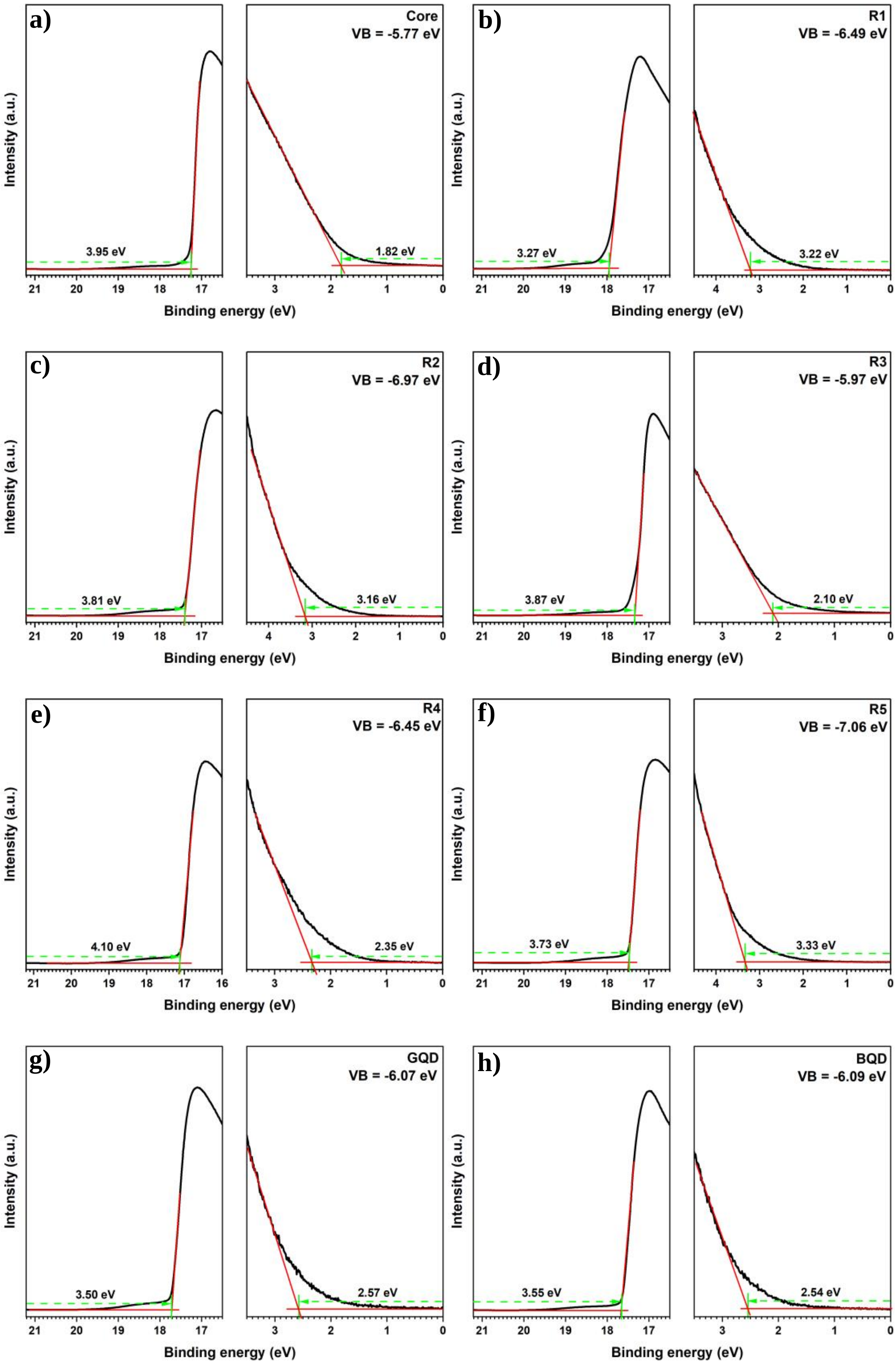

Figure S6. The UPS spectra of a) core, b)−e) **R1**−**R5**, g) **GQD** and h) **BQD**.

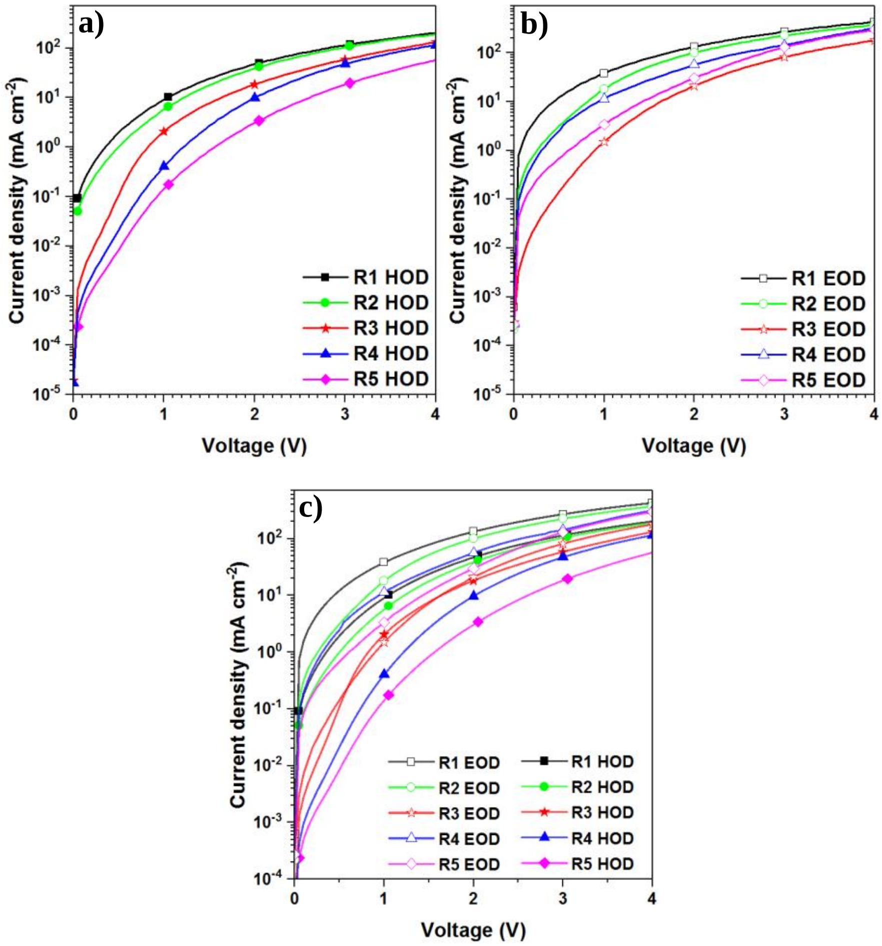

Figure S7. The current density *vs* voltage curves of single charge carrier based devices: a) hole-only devices (HODs), b) electron-only devices (EODs) and c) the comparison between the HODs and EODs based on red QDs.

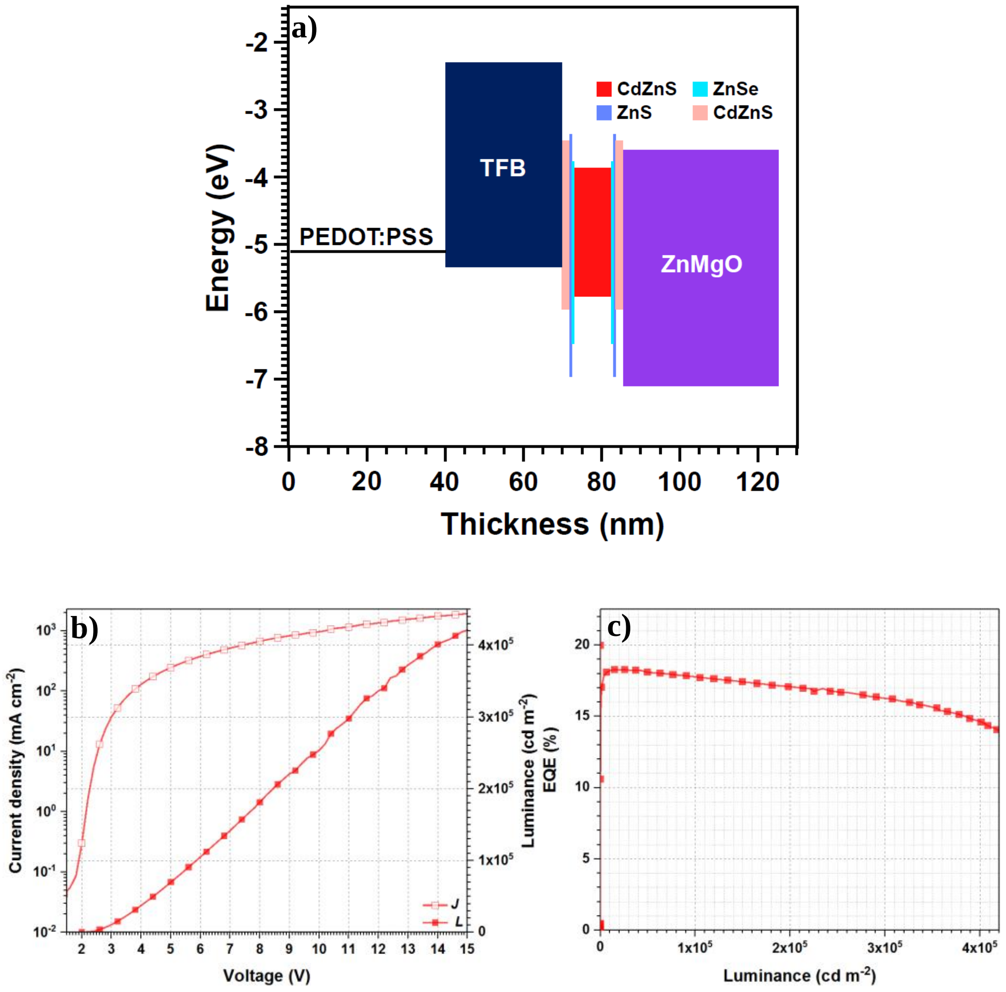

Figure S8. a) Energy diagram in **D3**, b) *J*−*V*−*L* and b) EQE−*L* curves of **D3** when the applied voltage is increased to 15 V.

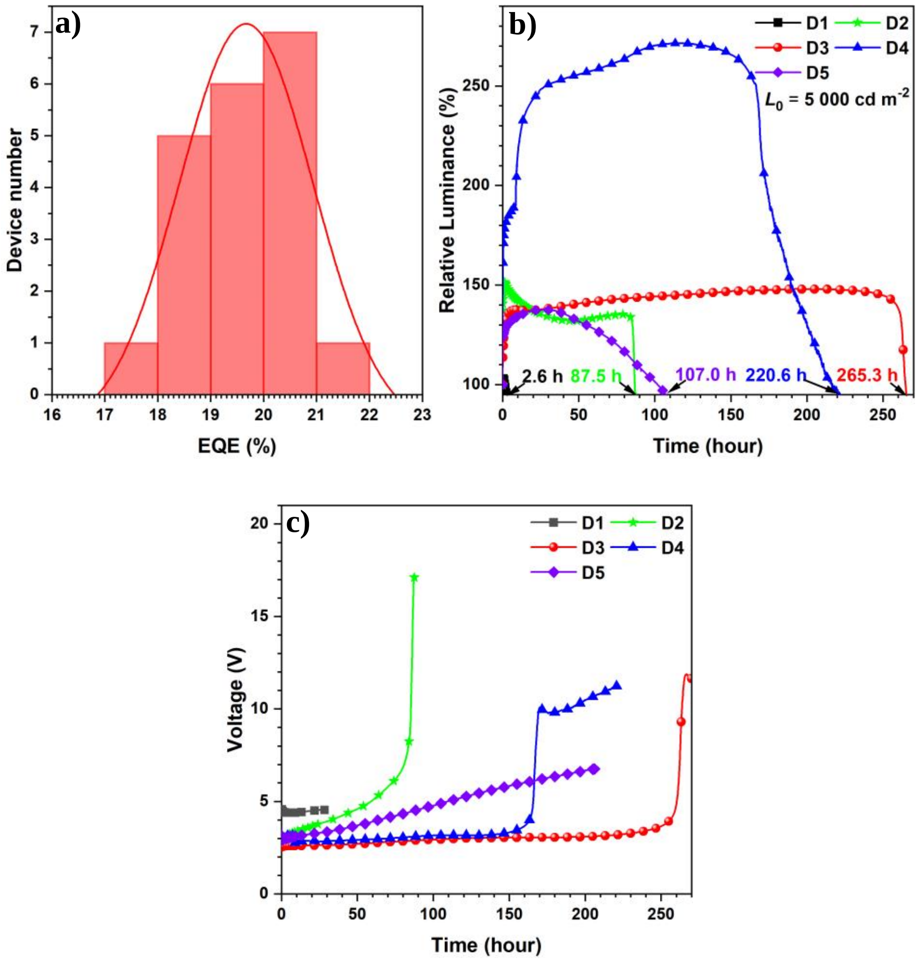

Figure S9. Stabilities of red QLEDs made by the CdZnS shell based QDs: a) EQE reproducibility, b) and c) operational lifetimes and the corresponding voltages for red QLEDs, respectively.

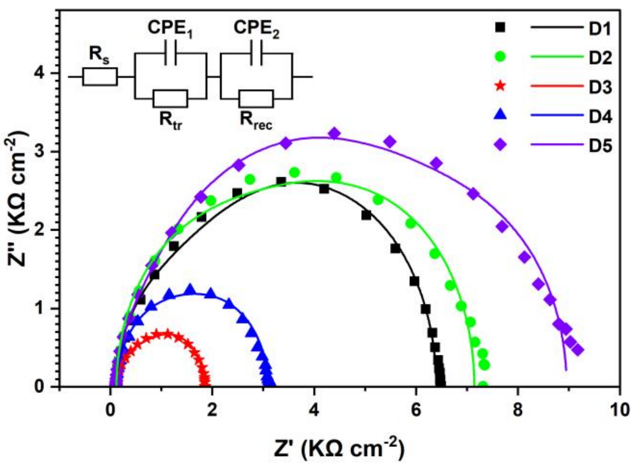

Figure S10. The measured (lines) and fitted (symbols) Nyquist plots of red QLEDs (inset: the equivalent circuit of these QLEDs).

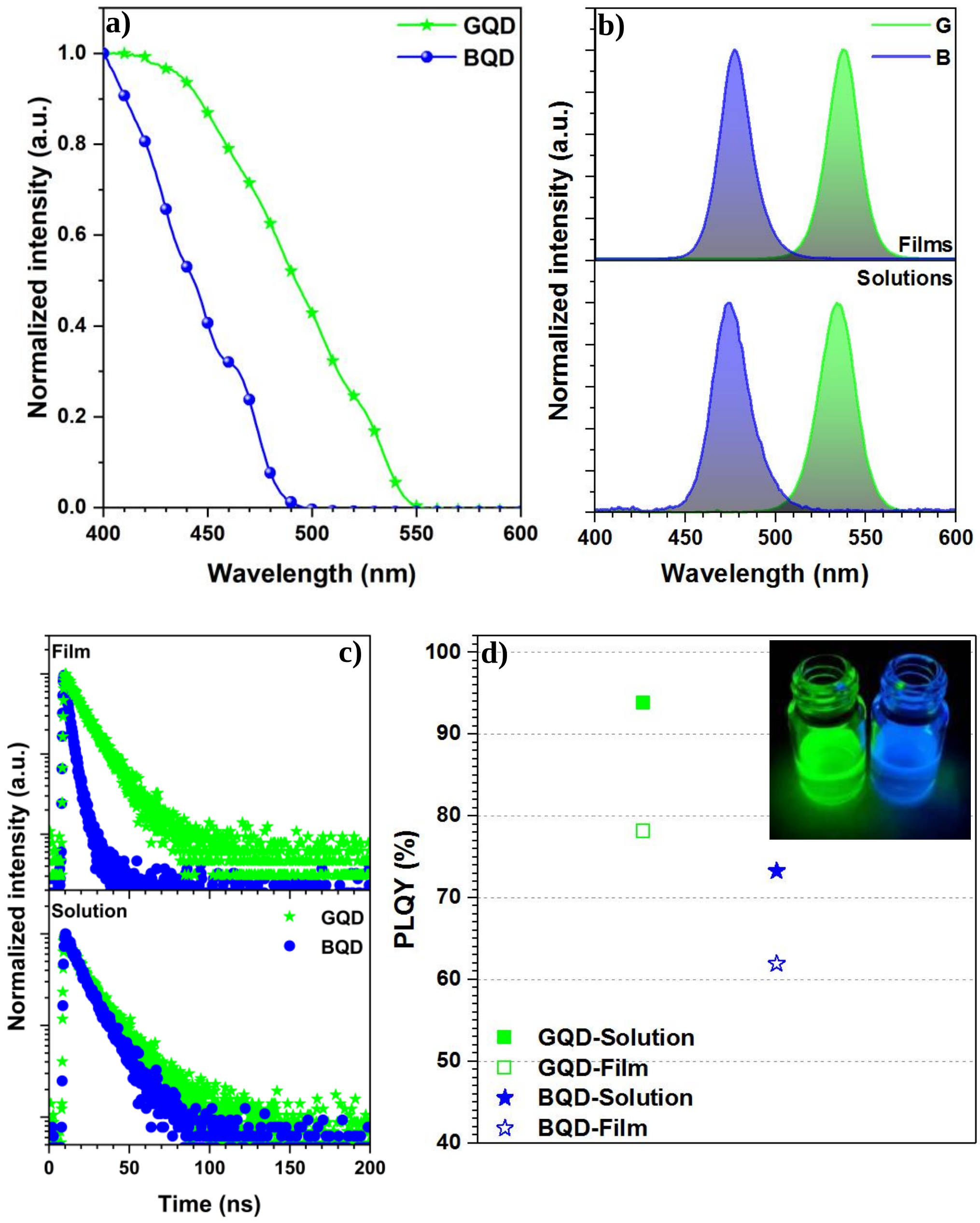

Figure S11. a),b) The UV-visible absorption and PL spectra for the green and blue QDs; c) PL decay curves and d) the PLQYs of the green and blue QD solutions and films (inset: the image of green and blue QD solutions under a 365 nm UV light).

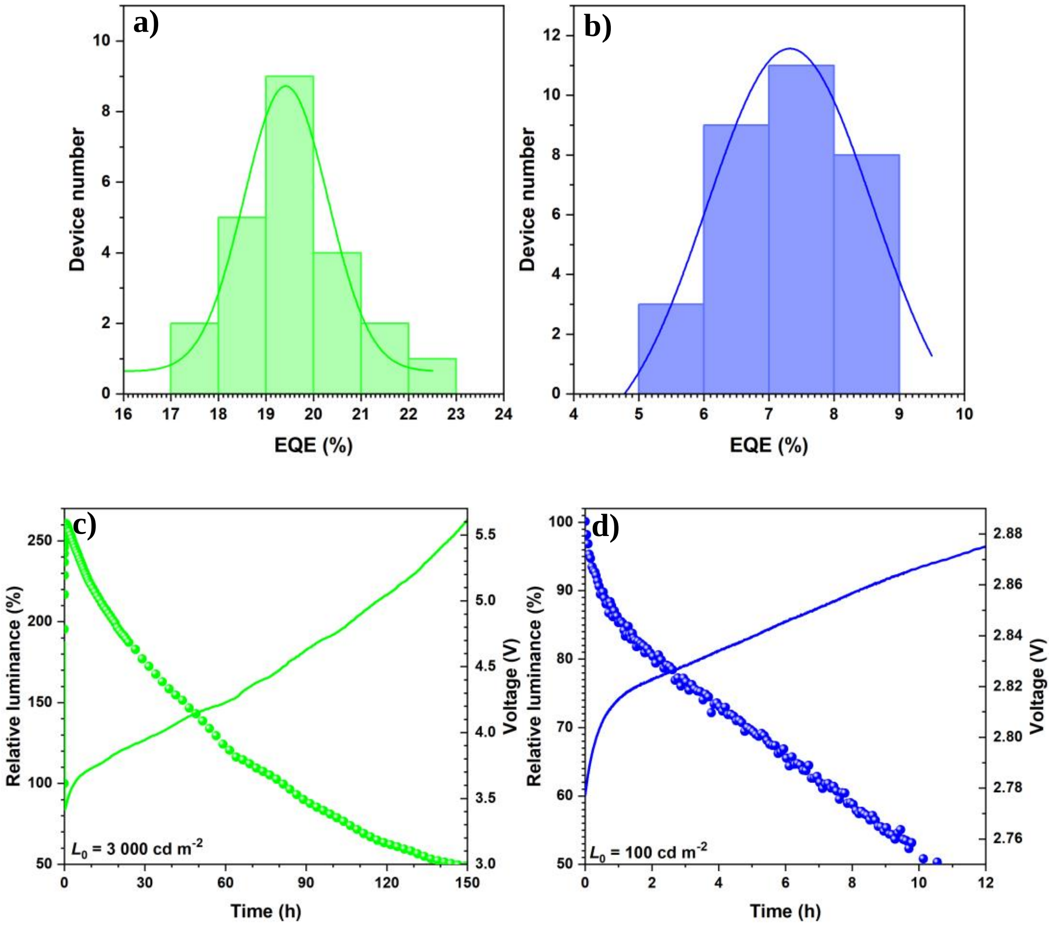

Figure S12. Stabilities of green and blue QLEDs: a) and b) EQE reproducibility; c) and d) operational lifetimes (symbols) and the corresponding voltages (lines) for green and blue QLEDs, respectively.

Table S1. The photophysical properties of QD solutions.

Solutions	PL (nm)	PLQY (%)	τ_1/A_1 (ns)/(%)	τ_2/A_2 (ns)/(%)	χ^2	τ[a)] (ns)
**Core (R)**	635	30.2	17.23/1.00	-	1.291	17.23
**R1**	620	37.9	17.38/1.00	-	1.113	17.38
**R2**	618	62.1	18.06/1.00	-	1.138	18.06
**R3**	622	94.4	22.28/1.00	-	1.090	22.28
**R4**	618	83.4	18.58/1.00	-	1.059	18.58
**R5**	619	77.9	19.39/1.00	-	1.028	19.39
**GQD**	537	93.8	10.24/37.24	22.05/62.76	1.075	19.50
**BQD**	477	73.3	11.25/83.88	24.12/16.12	1.012	15.00

[a)]The lifetimes (τ) of QDs calculated by the equation of $\tau = \sum A_i\tau_i^2/\sum A_i\tau_i$, where A_i is the amplitude of decay time constant (τ_i).

Table S2. The photophysical properties of QD films.

Films	PL (nm)	PLQY (%)	τ_1/A_1 (ns)/(%)	τ_2/A_2 (ns)/(%)	χ^2	τ (ns)
**Core (R)**	632	20.5	1.59/76.97	8.52/23.03	1.133	5.86
**R1**	619	29.9	2.32/71.89	8.53/28.11	1.073	5.98
**R2**	617	54.2	3.29/58.46	9.15/41.54	1.066	7.18
**R3**	620	83.7	13.61/1.00	-	1.036	13.61
**R4**	617	71.7	4.87/53.91	13.11/46.09	1.205	10.61
**R5**	618	70.2	7.59/87.10	19.47/12.90	1.191	10.86
**GQD**	534	78.1	5.80/66.14	15.35/33.86	1.168	11.29
**BQD**	474	61.9	2.79/80.04	7.99/19.96	1.039	4.96

Table S3. Fitting parameters of the EIS Nyquist plots for red QLEDs at their turn-on voltages.

Device	R_s (Ω cm^{-2})	R_{tr} (Ω cm^{-2})	CPE_1 (S Sec^n cm^{-2})	R_{rec} (Ω cm^{-2})	CPE_2 (S Sec^n cm^{-2})
**D1**	122	1,498	2.273×10^{-9}	4,776	4.281×10^{-9}
**D2**	121	2,948	1.891×10^{-9}	4,075	7.300×10^{-9}
**D3**	119	1,144	5.393×10^{-9}	582	1.901×10^{-9}
**D4**	122	1,580	5.978×10^{-9}	1,388	1.727×10^{-9}
**D5**	121	5,983	5.987×10^{-9}	2,602	2.055×10^{-9}

References

[1] O. Carion, B. Mahler, T. Pons, B. Dubertret, *Nat. Protocols* **2007**, *2*, 2383.

[2] B. O. Dabbousi, J. Rodriguez-Viejo, F. V. Mikulec, J. R. Heine, H. Mattoussi, R. Ober, K. F. Jensen, M. G. Bawendi, *J. Phys. Chem. B* **1997**, *101*, 9463.

[3] J. Zhou, M. Zhu, R. Meng, H. Qin, X. Peng, *J. Am. Chem. Soc.* **2017,** *139*, 16556.

[4] X. Jin, K. Xie, T. Zhang, H. Lian, Z. Zhang, B. Xu, D. Li, Q. Li, *Chem. Commun.* **2020,** *56*, 6130.

[5] Y. Altintas, U. Quliyeva, K. Gungor, O. Erdem, Y. Kelestemur, E. Mutlugun, M. V. Kovalenko, H. V. Demir, *Small* **2019,** *15*, 1804854.

[6] W. Wu, Z. Chen, Y. Zhan, B. Liu, W. Song, Y. Guo, J. Yan, X. Yang, Z. Zhou, W.-Y. Wong, *Adv. Mater. Interfaces* **2021**, *8*, 2100731.

[7] H.-W. Mo, Y. Tsuchiya, Y. Geng, T. Sagawa, C. Kikuchi, H. Nakanotani, F. Ito, C. Adachi, *Adv. Funct. Mater.* **2016**, *26*, 6703.
